# Recurrent Gastrointestinal Bleeding in a Patient With Severe Aortic Valve Stenosis: A Diagnosis of Heyde’s Syndrome

**DOI:** 10.7759/cureus.15442

**Published:** 2021-06-04

**Authors:** Adham E Obeidat, Jean Kim

**Affiliations:** 1 Internal Medicine, University of Hawaii, Honolulu, USA

**Keywords:** aortic stenosis, intestinal angiodysplasia, gastrointestinal bleeding, arteriovenous malformations, acquired coagulopathy, melena, aquired von willbrand disease

## Abstract

Heyde’s syndrome is defined as the coexistence of aortic valve stenosis (AS) and recurrent gastrointestinal (GI) bleeding from intestinal angiodysplasia (IA). Despite the fact that Heyde’s syndrome was first described decades ago, the association between AS and IA remains frequently unidentified, and thus, a high clinical suspicion is needed for its diagnosis. Here we present a case of a 60-year-old man with a history of severe AS, who presented with recurrent GI bleeding and was found to have multiple intestinal angioectasias on endoscopy.

## Introduction

The coexistence of aortic valve stenosis (AS) and recurrent gastrointestinal (GI) bleeding from intestinal angiodysplasia (IA) is known as Heyde’s syndrome [1,2]. This association was first described by Edward Heyde, MD, in 1958, when he reported 10 elderly patient cases with concurrent AS and GI bleeding [3]. Despite the recognition of Heyde’s syndrome for decades and the growing body of evidence behind its entity, the association between AS and IA remains frequently unidentified. Heyde’s syndrome can be complicated with high morbidity and mortality if it has not been diagnosed or managed properly, and thus, physicians need to be aware of this presentation. Here we present a case of a 60-year-old man with a history of severe AS, who presented with recurrent GI bleeding and found to have multiple intestinal angioectasias on endoscopy.

## Case presentation

This is a 70-year-old man with a past medical history of hypertension, hepatitis C infection, Barrett’s esophagus, a Zenker’s diverticulum, and severe AS, who presented to the emergency room (ER) complaining of new-onset melena and dyspnea. The patient reported melena starting one week prior to admission, but it was intermittently occurring for two months prior. The patient also reported progressive worsening of epigastric pain and nausea but no vomiting during this time. Furthermore, the patient had dyspnea and chest discomfort for two months, with associated dizziness. He denied any change in bowel habits, hematemesis, or bleeding from another site. He was admitted six months prior with the same complaints when a transthoracic echocardiogram (TTE) was performed and showed a normal left ventricular ejection fraction (LVEF) of 55%-60% and evidence of severe AS with an aortic valve area of 0.7 cm2, mean gradient 52 mmHg, and a peak aortic velocity of 4.4 m/s. At that time, an esophagogastroduodenoscopy (EGD) was completed which demonstrated Barrett's esophagus with high-grade dysplasia. Moreover, there was evidence of gastritis and a non-bleeding gastric ulcer with a clean base, which was biopsied, and it was negative for dysplasia or malignancy, as well as for Helicobacter pylori infection. Therefore, the patient was started on proton pump inhibitor (PPI) therapy. 

On admission, the physical examination was significant for a grade III systolic ejection murmur in the right second intercostal space. The remainder of the physical exam was unremarkable including the abdominal exam. Laboratory studies were significant for microcytic anemia; serum hemoglobin 5.9 g/dL, hematocrit 20.6%, and mean corpuscular volume (MCV) 76.3 fL. White blood cells (WBC) and platelet counts were within normal limits. The basic metabolic profile was unremarkable, while the liver function test showed a slightly elevated aspartate aminotransferase (AST) to 53 IU/L, but was otherwise normal. Lipase, urinalysis, and coagulation profile were all within normal limits.

**Figure 1:**
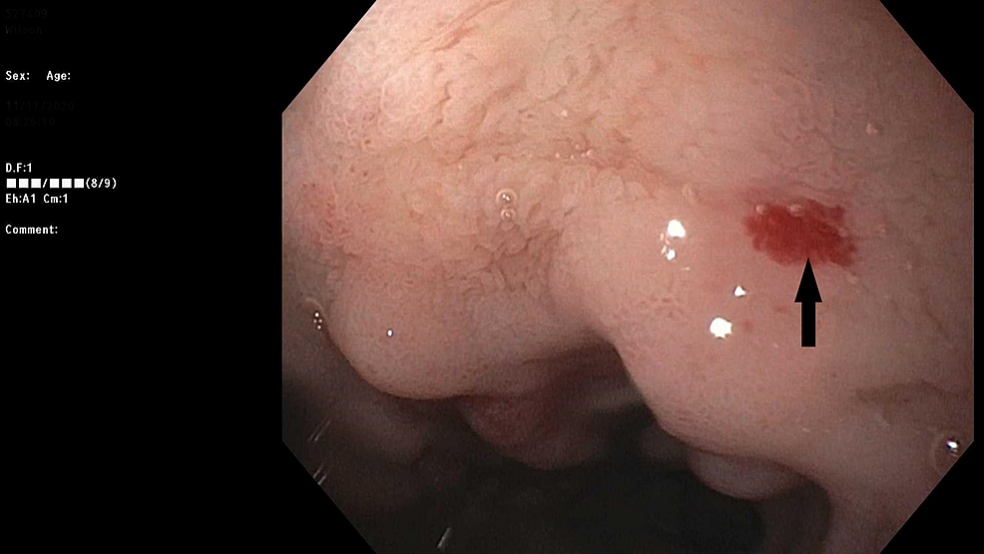
Endoscopic image shows duodenal angioectasia with stigmata of recent bleeding.

In the ER, the patient was given two units of packed red blood cells and was started on intravenous PPI. An EGD was repeated which showed three non-bleeding duodenal angioectasias (Figure 1), which were treated with electrical cauterization. A colonoscopy was recommended but the patient refused. Subsequently, the patient underwent a balloon valvuloplasty for his severe symptomatic AS. He was not deemed to be a candidate for surgical aortic valve replacement (AVR) given his social status of being homeless and was also not a candidate for trans-catheter aortic valve replacement (TAVR) as he would not be able to be started on dual anti-platelet therapy (DAPT) given his active GI bleeding.

**Figure 2:**
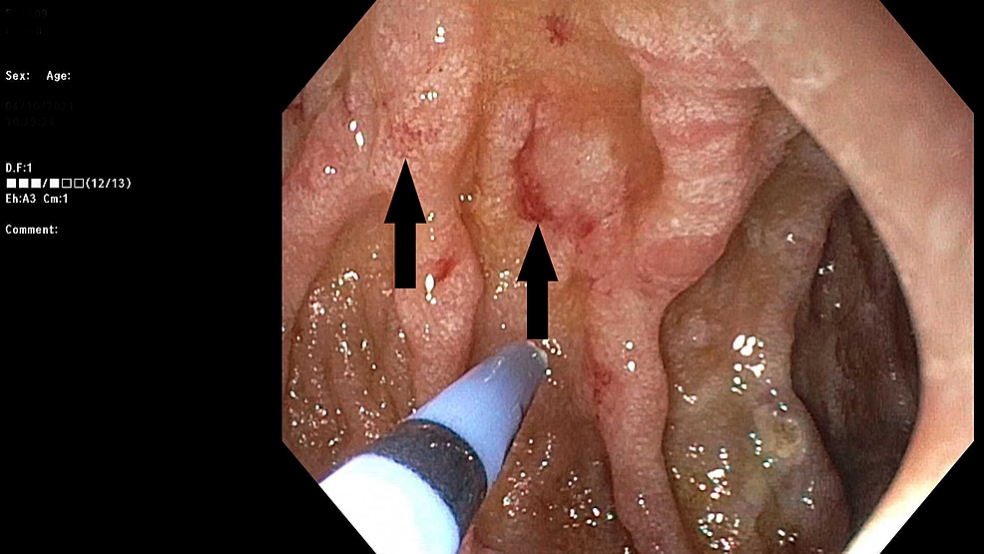
Multiple 2-4 mm angioctasias with stigmata of recent bleeding in the second part of the duodenum.

Upon discharge, the patient was admitted again multiple times with recurrent melena, dizziness, and acute blood loss anemia requiring blood transfusions. An EGD with push enteroscopy was repeated twice, which showed multiple duodenal and jejunal angiodysplastic lesions (Figure 2), which were treated with argon plasma coagulation. The patient was also evaluated by the cardiology service as an outpatient, and he underwent TAVR subsequently when his GI bleeding stopped. Aspirin was changed to Plavix accordingly, and the patient was continued on PPI. The patient has been doing well since the TAVR procedure, with no recurrent episodes of GI bleeding or anemia. The follow-up TTE after the TAVR showed a normal LVEF of 55%-60% and no evidence of AS (aortic valve area 2.2 cm^2^, mean gradient 11 mmHg, and a peak aortic velocity of 2.3 m/s).

## Discussion

Aortic stenosis and IA are common conditions found in the elderly; however, the two disorders are not frequently associated together. Heyde’s syndrome is a multiorgan system disorder that involves AS and GI bleeding secondary to IA [1,2]. Angiodysplasias are the most common vascular abnormalities of the GI tract and the second leading cause of lower GI bleeding in the elderly [1,4]. Moreover, around 1%-6% of hospital admissions for GI bleeding are attributed to IA [5]. Many studies were published regarding a possible association between AS and GI submucosal bleeding (i.e., arteriovenous malformations [AVM]), but to date, the association and the proposed pathophysiology of the entity remain controversial.

In a retrospective study by Oneglia et al., there was no increased prevalence of AS in patients with GI angiodysplasia [6]. Likewise, in a prospective controlled study by Bhutani et al., there was no increased incidence of AS in patients with a confirmed diagnosis of GI AVM [7]. Both studies, however, were limited by a small sample size of 83 and 40 total patients, respectively. On the other hand, a retrospective study by Batur et al. showed that the incidence of severe AS in patients with AVM was 4.1 times that in the general population [8].

Several hypotheses for the pathophysiology of Heyde’s syndrome have been proposed. The first theory suggests that the dilation of blood vessels induced by a chronic hypo-oxygenation of the microcirculation from AS can lead to a formation of angiodysplasia and subsequent GI bleed [9,10]. Another more prevailing theory suggests that von Willebrand factor (vWF), a multimeric glycoprotein that aids in primary hemostasis, becomes altered in its structure from the shear stress in AS and is more susceptible to proteolysis by ADAMTS13 [11,12]. This may lead subsequently to the development of von Willebrand syndrome type 2A (vWS-2A), an acquired coagulopathy, which helps explain the increased incidence of anemia and GI bleeding in patients with AS. Moreover, Vincentelli et al. concluded that vWF abnormalities are directly related to the severity of AS, and are improved by valve replacement [12].

Acquired vWS-2A can be diagnosed by an electrophoresis of vWF multimers [13]. Other tests such as vWF antigen levels and ristocetin cofactor activity may be normal in Heyde’s syndrome [1]. In this case, a defect in platelet aggregation can be proved by measuring closure times using collagen/ADP and collagen/epinephrine ratios [14]. Our patient’s coagulation profile and platelet count were normal; however, he did not have any of the diagnostic tests of the acquired vWS-2A.

The primary management of GI bleeding in Heyde’s syndrome consists of treating the AS [10]. Many case reports and case series have shown that GI bleeding was effectively controlled in patients who received AVR while most of those who instead received laparotomy with or without bowel resection did not [15-17]. Meanwhile, the treatment modalities utilized in von Willebrand diseases, such as desmopressin, octreotide, or supplementation of vWF or factor VIII, are usually ineffective for acquired vWF-2A [17,18]. Therefore, it is important to recognize and diagnose Heyde’s syndrome promptly and treat the aortic valvular abnormalities when possible, as these patients may not respond to other management modalities of GI bleeding.

Heyde’s syndrome is usually diagnosed in patients with comorbidities, which may put them at high risk for undergoing surgical AVR. Therefore, TAVR is considered a suitable treatment option in these patients. Sedaghat et al. showed that the restoration of the high-molecular-weight vWF multimer was similar in patients undergoing surgical AVR vs TAVR [19]. Our patient was considered to be at high risk for surgical AVR given his co-morbidities and social status; therefore, TAVR was pursued upon stabilization of his GI bleeding, with successful resolution of the severe AS, and no recurrent GI bleeding was reported since then.

## Conclusions

Heyde’s syndrome is often unidentified, and its pathophysiology remains controversial. The diagnosis of Heyde’s syndrome needs a high clinical suspicion, and it can be associated with high morbidity and mortality if it was not diagnosed and managed properly. Therefore, a prompt diagnosis of Heyde’s syndrome and treating AS with AVR when appropriate can help manage the recurrent GI bleeding refractory to other treatment modalities. Our patient was admitted multiple times with recurrent GI bleeding before the diagnosis of Heyde’s syndrome was made, and he underwent TAVR with subsequent resolution of his symptoms.
